# Case report: Virus-induced hemophagocytic lymphohistiocytosis in a patient with APECED

**DOI:** 10.3389/fped.2023.1086867

**Published:** 2023-02-15

**Authors:** Oksana Boyarchuk, Olha Dyvonyak, Tetyana Hariyan, Alla Volokha

**Affiliations:** ^1^ Department of Children's Diseases and Pediatric Surgery, I.Horbachevsky Ternopil National Medical University, Ternopil, Ukraine; ^2^ Municipal Children's Hospital, Ternopil, Ukraine; ^3^ Department of Pediatrics, Pediatric Infectious Diseases, Immunology and Allergology, Shupyk National Healthcare University of Ukraine, Kyiv, Ukraine

**Keywords:** AIRE, APECED, APS-1, COVID-19, macrophage activation syndrome, haemophagocytic lymphohistiocytosis

## Abstract

Autoimmune polyendocrinopathy-candidiasis-ectodermal dystrophy (APECED), also known as autoimmune polyglandular syndrome type 1 (APS-1) is a rare autosomal recessive inborn error of immunity (IEI), which is accompanied by immune dysregulation. Hypoparathyroidism, adrenocortical failure and candidiasis are its typical manifestations. Here we report about recurrent COVID-19 in a 3-year-old boy with APECED, who developed retinopathy with macular atrophy and autoimmune hepatitis after the first episode of SARS-CoV-2 infection. Primary Epstein-Barr virus infection and a new episode of SARS-CoV-2 infection with COVID pneumonia triggered the development of severe hyperinflammation with signs of hemophagocytic lymphohistiocytosis (HLH): progressive cytopenia (thrombocytopenia, anemia, lymphopenia), hypoproteinemia, hypoalbuminemia, high levels of liver enzymes, hyperferritinemia, increased triglycerides levels; and coagulopathy with a low level of fibrinogen. Treatment with corticosteroids and intravenous immunoglobulins did not lead to a significant improvement. The progression of HLH and COVID-pneumonia resulted in a fatal outcome. The rarity and varied presentation of the HLH symptoms led to diagnostic difficulties and diagnosis delay. HLH should be suspected in a patient with immune dysregulation and impaired viral response. Treatment of infection-HLH is a major challenge due to the difficulties in balancing immunosuppression and management of underlying/triggering infection.

## Introduction

Autoimmune polyendocrinopathy-candidiasis-ectodermal dystrophy (APECED), also known as autoimmune polyglandular syndrome type 1 (APS-1) is a rare autosomal recessive inborn error of immunity (IEI), which is accompanied by immune dysregulation (1, 2). APECED is caused by a mutation in the Autoimmune Regulator (*AIRE*) gene and characterized by chronic mucocutaneous candidiasis (CMC) and multisystem autoimmunity. Other frequently reported symptoms include hypoparathyroidism and adrenocortical failure (1), and together with candidiasis they form a classic triad (2). The presence of at least two signs of the triad is required for diagnosis.

Other organs and systems can be affected with various frequency and these most often involve the endocrine organs and autoimmunity, such as hypothyroidism, type 1 diabetes mellitus, pernicious anemia, alopecia, vitiligo, malabsorption, Sjogren-like syndrome, pneumonitis, and hepatitis (2–5).

Candidiasis of the skin or mucous membranes is usually one of the first symptoms; most frequently it presents as a recurrent oral candidiasis or diaper dermatitis (6). The frequency of APECED-associated hepatitis ranges from about 10% in the European population to 42% in American cohorts (2), and in a third of the patients in the American cohort, hepatitis symptoms preceded the other classic APECED criteria.

Ocular manifestations are also reported in patients with APECED and are associated with a poor prognosis (6–9). Keratopathy and retinopathy are the most frequent among ocular abnormalities (6–8). Vision loss due to macular atrophy had also been detected. Specific autoantibodies against corneal and/or retinal antigens are the main causes responsible for the ocular manifestations in patients with APECED (8). Moreover, there is no correspondence between the severity of eye symptoms and other systemic manifestations.

Besides CMC, patients with APECED have a high susceptibility to some other specific infections that are mediated by anti-cytokine autoantibodies and/or Tcell driven autoimmune tissue injury (10). High titers of autoantibodies to IFN-α present in almost every patient with APECED result the severe course of viral infections in this cohort of patients (10, 11). This has become especially relevant during the COVID-19 pandemic, because the presence of autoantibodies to type I IFNs underlies the severe COVID-19 pneumonia in patients with APECED (12–14).

Here we report about recurrent COVID-19 in a 3-year-old boy with APECED, who developed retinopathy with macular atrophy and autoimmune hepatitis after the first episode of SARS-CoV-2 infection. Primary Epstein-Barr virus (EBV) infection and a new episode of SARS-CoV-2 infection with COVID pneumonia triggered the development of severe hyperinflammation with signs of hemophagocytic lymphohistiocytosis (HLH), which led to a fatal outcome.

## Case report

A 3-year-8-month-old boy was admitted to the hospital in January, 2022 with complaints of fever up to 39°C, runny nose, cough, reduced appetite, irritability.

The first symptoms appeared 5 days before the admission. He was treated for an upper respiratory infection, received symptomatic and antibiotic therapy. His condition did not improve, leading to his hospitalization.

The patient's history showed that he was the first child of non-consanguineous parents. His weight at birth was 3,080 g. Vaccinations were carried out according to the schedule. On the 11th day after the first measles, mumps, and rubella (MMR) vaccination, the boy developed symptoms of measles (fever, rash), for which he was hospitalized.

In September 2020, the boy had COVID-19 after a contact with sick family members. The course of the disease was mild, accompanied by a cough for several days. However, a few days after COVID-19, the mother noticed a rapid progressive deterioration of the child's vision, up to a complete blindness. The boy was examined for this complaint in several clinics and was diagnosed with retinopathy and secondary macular dystrophy of unknown origin. During a hospitalization in November 2020, additional tests to clarify the diagnosis revealed acute otitis media, candidiasis of the oral mucosa and an increase in the levels of liver enzymes: aspartate aminotransferase (AST) up to 228.6 U/L, alanine aminotransferase (ALT) up to 566.5 U/L, and lactate dehydrogenase (LDH) up to 376.6 U/L (see Table 2 for the reference ranges). The levels of bilirubin, alkaline phosphatase, albumin, gamma-glutamyl transferase (GGT) were within normal limits. Leukocytosis of 15.7 10^9^/L was detected, while other values of the complete blood count (CBC), including the level of platelets, as well as the coagulogram indexes were within normal limits. Tests were carried out to exclude viral hepatitis B and C, cytomegalovirus (CMV) infection, Epstein-Barr virus (EBV) infection, toxoplasmosis, rubella, and HIV infection. IgM and/or IgG antibodies to the indicated pathogens were negative, except antibodies to rubella (high titer of IgG to rubella antibodies), which could indicate that the patient had rubella or the presence of post-vaccination antibodies.

Given the development of measles after MMR vaccination, the signs of liver damage, and moderate manifestations of candidiasis, a congenital pathology, such as inborn errors of immunity, was suspected. Immunological examination revealed no significant abnormalities, except for a slightly increased level of IgG (Table 1). Whole exome sequencing (WES) detected a mutation in the *AIRE* gene, which was confirmed by gene sequencing. Two homozygous pathogenic variants, c.769C > T (*p*.Arg257 Ter), were identified in *AIRE*. Among the markers of autoimmune hepatitis, only liver cytosolic antigen (LC-1, IgG antigen) was positive.

**Table 1:** Immunological parameters of the patient.

Parameter	17.03.2021/2 years 10 mos	Reference range
CD3+, %; cell/µl	60; 4200	66–73; 1610–4230
CD4+, %; cell/µl	30; 2100	32–43; 900–2860
CD8+, %; cell/µl	28; 1960	25–34; 630–1910
CD4/CD8, %; cell/µl	1.1	1.1–1.4
CD19+, %; cell/µl	16; 1120	16–24; 700–1300
CD56 + CD16+	7; 490	7–15; 100–400
IgG, g/L	19.9	7.23–16.82
IgA, g/L	1.18	0.69–3.82
IgM, g/L	0.94	0.63–2.7

Autoimmune hepatitis and oropharyngeal candidiasis were diagnosed as signs of APECED. The patient received azathioprine, oral glucocorticoids with a gradual dose reduction and subsequent withdrawal, antifungal therapy, calcium and vitamin D. On the background of treatment, the levels of liver enzymes have normalized. Glucocorticoids were discontinued and the patient continued azathioprine intake.

In admission to the hospital in January 2022, the patient's condition was estimated as moderate. His weight was 15 kg (*z* −0.32), height 94 cm (*z* −1.36). The child was irritable. Slight swellings on the face, cracks on the lips were observed. The throat was hyperemic. The heart rate was 130 beats per minute, respiratory rate - 26 breaths per minute, and oxygen saturation - 96%–98%. Vesicular breathing was heard over the thorax. The liver protruded 2 cm from the edge of the right costal arch, the spleen was located along the edge of the left costal arch.

Considering prolonged fever, hyperemic throat, increased incidence of SARS-CoV-2 infection, associated with the Omicron variant COVID-19 was suspected, but the PCR test for COVID-19 was negative on admission. CBC revealed leukopenia, moderate lymphopenia, thrombocytopenia, a pronounced shift of the indices to the left (33% of band neutrophils), and elevated erythrocyte sedimentation rate (ESR) (Table 2). Biochemical tests showed hypoproteinemia, hypoalbuminemia, hyperbilirubinemia due to direct bilirubin, high levels of ALT, AST, GGT, LDG, and a reduced calcium level (Table 2). CRP was elevated (50.3 mg/L), while procalcitonin was normal (0.13 ng/ml). A high level of ferritin was detected (1,297 ng/ml). Chest x-ray on admission showed no pathological changes. Echocardiographic findings did not reveal cardiac dysfunction, pericarditis, valvulitis, or coronary abnormalities.

**Table 2:** Clinical data, laboratory test results and treatment of the patient.

Indicator/Date		23.01	25.01	26.01	27.01	28.01	29.01	30.01	31.01	Reference range
Fever, °С		38.8	37.2	38.6	38.8	37.4	38.3	37.8	37.6	
SARS-CoV-2	PCR	neg	neg		**pos**	** **				
	Serology (IgM + IgA)				**pos**	** **				
	Antigen test	neg				** **				
EBV IgM					**pos**					
WBC, 10^9^/L		**3** **.** **36**	**3**.**21**		**2**.**94**	3.44	**1**.**87**	**1**.**72**	**1**.**80**	4.0–10.0
Neutrophils, 10^9^/L		1.54	1.93		1.53	2.40	**1**.**30**	**1**.**08**	**1**.**02**	1.5–7.0
Lymphocytes, 10^9^/L		**1** **.** **49**	**1**.**15**		**1**.**29**	**0**.**96**	**0**.**54**	**0**.**55**	**0**.**68**	2.0–6.5
Platelets, 10^9^/L		**83**	**84**	** **	**60**	**25**	**15**	**12**	**19**	150–400
Hemoglobin, g/L		120	126	** **	**104**	**101**	**84**	**74**	**62**	120–140
Ferritin, ng/mL				**1297**						22–350
CRP, mg/L		** **	** **	**50** **.** **3**		–	**42**.**9**		–	<5
LDH,U/L		** **	** **	**1474**		**1588**	** **		**1214**	120–300
AST, U/L		** **	** **	**495**		**930**	**256**		**247**	<40
ALT, U/L		** **	** **	**245**		**197**	**168**		**151**	<37
GGT, U/L		** **	** **	**101**		** **	**104**		**127**	<55
Bilirubin, µmol/L		** **	** **	**38** **.** **3**	** **	**96**.**5**	**77**.**9**		**46**.**8**	<21
TGL, mmol/L		** **	** **	** **	** **	**3**.**28**	** **		**3**.**93**	<1.71
Albumin, g/L		** **	** **	**25** **.** **6**	** **	**23**.**8**	** **		**26**.**1**	35–52
Total protein, g/L		** **	** **	**53**	** **	**45**	**50**		65	60–83
Creatinine, µmol/L		** **	** **	33	** **	32	43.4		49	70–100
D-dimer, ng/ml		** **	** **		** **		1810			<250
Fibrinogen, g/L		** **	** **		** **	**0**.**44**	–	**0**.**3**	**0**.**46**	2.0–4.0
PTT, sec		** **	** **	** **	** **	27.8	**14**.**4**	29.2	**52**.**5**	25–38
PT, sec		** **	** **	** **	** **	16.0	**19**.**8**	14.6	14.5	13–17
INR		** **	** **	** **	** **	**1**.**35**	**1**.**65**	0.91	1.23	0.8–1.2
Prothrombin, %		** **	** **	** **	** **	71.4	**56**.**7**	**14**.**6**	79.4	70–100
Treatment		Antibiotic + symptomatic therapy								
		** **	** **	** **	** **	Pulse GC + IVIG			GC	
		** **	** **	** **	** **		Cryoplasma			

PCR, polymerase chain reaction; WBC, white blood count; CRP, C-reactive protein; LDH- lactate dehydrogenase; AST, aspartate aminotransferase; ALT, alanine aminotransferase; GGT, gamma-glutamyl transferase; TGL, triglycerides; PTT, partial thromboplastin time; PT, prothrombin time; INR, international normalized ratio; GC, glucocorticoids; IVIG, intravenous immunoglobulin. Indicators that do not fall within the normal range are highlighted in bold.

Given the negative PCR test for COVID-19, an examination was conducted to identify other causes of hepatitis. Hepatitis B and C markers (HBsAg, anti HCV IgM) were negative. However, a high titer of IgM antibodies to the Epstein-Barr virus (55.68) was observed with a normal IgG indicator to the Epstein-Barr virus, 3.66 (reference <9). On the 4th day after admission (9th day after the onset of symptoms), a positive serology result for СОVID-19 was obtained: IgM + IgA antibodies to SARS-CoV-2 were positive (64.38; reference range 6–8); positive PCR test for COVID-19 was also obtained.

Prescribed symptomatic treatment and antibiotic therapy did not improve the patient's condition. The fever was maintained, oral candidiasis appeared. Hepatosplenomegaly increased (liver + 4 cm, spleen + 2 cm). Oxygen saturation decreased to 90%–92%.

Multisystem inflammatory syndrome in children (MIS-C), associated with COVID-19 was suspected considering hyperinflammation that presented as fever and elevated markers of inflammation (CRP, ESR, ferritin, LDH, lymphocytopenia, hypoalbuminemia).

However, lack of clinical signs of multisystem involvement according to WHO case definition of MIS-C (15), coagulopathy with a low level of fibrinogen, normal procalcitonin, the absence of neutrophilia and the development of neutropenia, increased triglycerides levels, alternative plausible diagnoses listed in the CDC case definition of MIS-C (16) more support to viral related secondary HLH or macrophage activation syndrome (MAS).

On the 5th day, due to the lack of positive dynamics and worsening symptoms, the child was transferred to the intensive care unit. Oxygen therapy, intravenous immunoglobulin (2 g/kg over 3 days), intravenous pulse methylprednisolone (10 mg/kg/day N 3), cryoplasma (10 ml/kg/day), and antifungal therapy (fluconazole 6 mg/kg/day) were prescribed, and antibiotic therapy (cefepime 100 mg/kg/day) was continued.

Despite the treatment, slight positive dynamics were observed only in some biochemical indicators (bilirubin, ALT, AST) and indicators of the coagulogram (Table 2). However, anemia, thrombocytopenia, leukopenia, and lymphopenia have exacerbated, and hypofibrinogenemia persisted (Table 2). On repeated chest x-ray, bilateral polysegmental pneumonia and right-sided exudative pleurisy were detected. Antibiotic therapy was changed to meropenem (60 mg/kg/day), additional cryoplasma (10 ml/kg/day), was prescribed. The boy was placed on a ventilator, but the escalating multiple organ failure led to the child's death.

## Discussion

The peculiarity of the presented case is a reсurrent COVID-19 infection complicated by HLH with a fatal outcome in an immunocompromised child with APECED. The first episode of COVID-19 in September 2020 had a mild course, but led to the manifestation of the symptoms of IEI. While these symptoms (retinopathy, hepatitis), except for the moderate mucosal candidiasis, are not included in the triad of classic APECED symptoms, taken together with the reaction to the live vaccine, it was possible to suspect immunodeficiency.

Immunosuppressive therapy led to stabilization of the hepatitis, but the eye symptoms had no positive dynamics. A second episode of COVID-19 occurred in January 2022. The course presented with prolonged, treatment resistant fever for 2 weeks, progressive cytopenia (thrombocytopenia, anemia, lymphopenia), hypoproteinemia, hypoalbuminemia, signs of active hepatitis (hyperbilirubinemia due to the direct fraction, high levels of liver enzymes), hyperferritinemia, increased triglycerides level; coagulopathy with a low level of fibrinogen. It was only possible to confirm COVID-19 on the 9th day after the onset of symptoms. Later, the signs of pneumonia arose, which was confirmed by x-ray. The patient met some criteria of MIS-C, related to SARS-CoV-2 infection, however, a low level of fibrinogen, the absence of neutrophilia and the development of neutropenia, increased triglycerides levels were more consistent with viral related secondary HLH.

Another feature of current case is the presence of a mixed infection: an active EBV infection was detected (by high positive IgM antibodies) along with COVID-19. The absence of IgG to EBV may indicate a primary infection that affected the course of COVID-19 and was also a likely trigger for hyperinflammation and the development of HLH/MAS.

In general, COVID-19 in patients with APECED has a severe course, due to the presence of antibodies to type I IFN in such patients, which has been confirmed by several recent studies (10–14). Autoantibodies to type I IFNs affect the severity of the course of COVID-19 in the general population and account for ∼20% of COVID-19 deaths (17). Neutralizing autoantibodies to type-I IFNs in patients with critical COVID-19 pneumonia are associated with delayed time to viral clearance (18). Unfortunately, we could not determine the presence of antibodies to type I IFN in our patient, although other studies indicate a high titer of them in nearly 100% of patients with APECED (11, 12). The involvement of other bronchial antigens, such as BPIFB1 and potassium regulator KCNRG was confirmed in the development of autoimmune pneumonitis in patients with APECED and some of lung epithelial self-antigens remain undetected (10, 19).

The other potential mechanism of severe COVID-19 in patients with APECED may be associated with impaired negative selection of autoreactive T-cells in connection with AIRE deficiency (10). These T-cells infiltrate different organs, including lungs and spleen, causing organ damage and dysfunction, and predisposing to secondary invasive bacterial infections (10).

In our opinion, the cause of death in the presented case was not only COVID pneumonia, but to a large extent also HLH. The pathophysiological basis of HLH/MAS is pathological immune activation, or the so-called cytokine storm (20, 21). HLH/MAS is characterized by fever, variable hepatosplenomegaly, high levels of CRP and ferritin, hemophagocytosis, cytopenias (including pancytopenia), and coagulopathy associated with liver pathology and disseminated intravascular coagulation (20). Initially, the MAS terminology was used in rheumatology regarding systemic juvenile idiopathic arthritis (sJIA) and adult-onset Still disease. It also corresponds to the secondary (acquired) HLH in autoimmune, oncological diseases or in combination with infections or other diseases (20), whereas primary HLH was defined as a pediatric monogenic immunodeficiency state with hyperinflammation (20).

Viral infections are the most common triggers of HLH/MAS in children (20–22). Among them, EBV is the most frequently identified, followed by CMV (17–19). However, other bacterial, parasitic, and fungal infections can also trigger HLH/MAS (20, 21).

The issue of cytokine storm and HLH became very relevant during the COVID-19 pandemic. Hyperferritinemia and coagulopathy associated with SARS-CoV-2 infection have been linked to a HLH -type phenotype, but the incidence of classical HLH/MAS in patients with COVID-19 is not well described (20).

HLH also occurs in various groups of immunocompromised patients, including those with IEI (21). Patients with severe combined immunodeficiency, Omenns syndrome, severe DiGeorge syndrome, Wiskott-Aldrich syndrome, X-linked agammaglobulinemia, chronic granulomatous disease, autoimmune lymphoproliferative syndrome have all been reported to present with HLH (23). Patients with IEI complicated by HLH often have severe, unresolved infections (21, 24). To the best of our knowledge, the reported case is the first patient with APECED who developed HLH. Unfortunately, combined HLH and COVID pneumonia have caused the patient's death.

The development of hyperinflammation in COVID-19 pneumonia can mimic signs of HLH. McGonagle et al. (20) point out the main differences between hyperinflammation in COVID-19 and MAS. With COVID-19 pneumonia, there is a localized tissue-specific cytokine response with the development of “local cytokine flooding”, while MAS is characterized by systemic macrophage activation and a global cytokine storm (20). In addition, concentrations of cytokines and ferritins in patients with COVID-19 pneumonia are lower than in patients with MAS.

In this case the potential presence of autoantibodies to type I IFNs might impair immune response, which led to a massive cytokine release syndrome with the development of systemic hyperinflammation. Impaired negative selection of autoreactive T-cells may have also contributed to cytokine storm and organs injury. Prolonged viral infection and antigenic stimulation led to the activation of T cells and macrophages, as well as the massive release of pro-inflammatory cytokines (25).

The HLH 2004 (26) and MAS 2016 (27) diagnostic criteria are summarized in Table 3. Symptoms of the patient fully correspond to these criteria, including 5 out of 8 criteria of HLH 2004 in addition to the presence of fever, hyperferritinemia and all other 4 criteria of MAS.

**Table 3:** Compliance of the patient's symptoms with the classification criteria for HLH 2004 and MAS 2016.

Criterion	HLH 2004	MAS 2016
Clinical criteria	**Fever**	**Fever**
	**Splenomegaly**	
**Cytopenias (affecting ≥2 of 3 lineages in the peripheral blood):**		
Hemoglobin, g/L	**<90**	
Platelets, cells/µl	**<100,000**	**≤180,000**
Neutrophils, cells/µl	<1000	
**Hypertriglyceridemia and/or hypofibrinogenemia:**		
Triglycerides, mmol/L	**≥3** **.** **0**	**>1.76** (156 mg/dl)
Fibrinogen, g/L	**≤1** **.** **5**	**≤3** **.** **6**
Ferritin, ng/ml	**≥500**	**>684**
AST, U/L	** **	**>48**
Hemophagocytosis in bone marrow or spleen or lymph nodes	**+**	** **
NK-cell activity	Low or absent	** **
Soluble CD25 (i.e., soluble IL-2 receptor), U/ml	>2,400	** **
Diagnosis	**Five** out of the eight criteria	**A febrile patient** with known or suspected JIA **plus ferritin,** **plus any two other laboratory criteria**

The symptoms present in the patient are highlighted in bold.

HLH/MAS treatment also remains a challenging issue. Immune dysregulation is the central problem in both APECED and HLH, therefore immunosuppressive therapy should be the priority (20, 21). The patient received pulse therapy with methylprednisolone and intravenous immunoglobulins in a suppressive dose, but this did not lead to a significant improvement of his condition. Anti-cytokine therapies, including anti-interleukin-1 (IL-1), IL-6, IL-18, interferon-*γ*, and janus kinase (JAK) could have been used as the second-line drugs in the treatment of the patient (20, 25, 28). Another drug has been reported to improve the clinical symptoms of the disease as a part of a complex treatment of Epstein Barr virus induced HLH is rituximab (29).

The verifiable presence of autoantibodies to type I IFNs in patients with APECED also requires the prescription of immunosuppressive therapy or other strategies to overcome the effect of autoantibodies to type I IFNs (12–14).

The other strategy is supporting immune response against SARS-CoV-2 virus using monoclonal antibodies to the SARS-CoV-2 spike protein (14), which block entry of SARS-CoV-2 in host cells Prescription of bamlanivimab and etesevimab for two siblings with APECED allowed to prevent invasive ventilatory support, admission to the intensive care, and death of these patients (14).

Thus, the initial symptoms of HLH/MAS are nonspecific and may overlap with other infectious, inflammatory and oncohematology conditions (21), especially in the COVID-19 era. Hyperinflammation in patients with COVID-19, including MIS-C can mimic and overlap with the signs of HLH. Rarity and diversity of the HLH/MAS symptoms led to diagnostic difficulties and diagnosis delay (21). Patients with IEI, especially with immune dysregulation are at high risk of HLH.

Rising awareness of symptoms of IEI, cytokine storm and HLH/MAS should improve their recognition (21, 30, 31) and accelerate the administration of appropriate treatment. However, some authors (21) alert about the risk of misdiagnosis and inappropriate treatment of HLH-mimicking conditions.

The limitation of this study is the impossibility to establish a definitive cause-effect relationship. The lack of certain examinations (EBV-DNA, cytokine levels) and the post-mortem diagnosis did not allow to fully determine the degree of damage to different organs and tissues. The short period between the diagnosis and death of the child did not permit to use the entire spectrum of treatment possibilities.

Thus, HLH should be suspected in a patient with immune dysregulation and impaired viral response. Treatment of infection-HLH is a major challenge due to the difficulties in balancing immunosuppression and management of underlying/triggering infection.

## Data Availability

The original contributions presented in the study are included in the article/Supplementary Material, further inquiries can be directed to the corresponding author.
